# Cold Atmospheric Plasma and Gold Quantum Dots Exert Dual Cytotoxicity Mediated by the Cell Receptor-Activated Apoptotic Pathway in Glioblastoma Cells

**DOI:** 10.3390/cancers12020457

**Published:** 2020-02-16

**Authors:** Nagendra Kumar Kaushik, Neha Kaushik, Rizwan Wahab, Pradeep Bhartiya, Nguyen Nhat Linh, Farheen Khan, Abdulaziz A. Al-Khedhairy, Eun Ha Choi

**Affiliations:** 1 Plasma Bioscience Research Center/Applied Plasma Medicine Center, Department of Electrical and Biological Physics, Kwangwoon University, Seoul 01897, Korea; pradeepindian65@gmail.com (P.B.); nhatlinhusth@gmail.com (N.N.L.); ehchoi@kw.ac.kr (E.H.C.); 2 Department of Laboratory Medicine, College of Medicine, Hanyang University, Guri 11923, Korea; neha.bioplasma@gmail.com; 3 Zoology Department, College of Science, King Saud University, Riyadh 11451, Saudi Arabia; kedhairy@yahoo.com; 4 Chair for DNA Research, King Saud University, Riyadh 11451, Saudi Arabia; 5 Laboratory of Plasma Technology, Institute of Materials Science, Vietnam Academy of Science and Technology, 18 Hoang Quoc Viet, Hanoi 100000, Vietnam; 6 Chemistry Department, Faculty of Science, Taibah University, Yanbu 42353, Saudi Arabia; khanfarheenchem@gmail.com

**Keywords:** gold quantum dots, plasma, cancer, nanomaterials, cellular uptake, invasiveness

## Abstract

Brain cancer malignancies represent an immense challenge for research and clinical oncology. Glioblastoma is the most lethal form of primary malignant brain cancer and is one of the most aggressive forms commonly associated with adverse prognosis and fatal outcome. Currently, combinations of inorganic and organic nanomaterials have been shown to improve survival rates through targeted drug delivery systems. In this study, we developed a dual treatment approach using cold atmospheric plasma (CAP) and gold quantum dots (AuQDs) for brain cancer. Our results showed that CAP and AuQDs induced dual cytotoxicity in brain cancer cells via Fas/TRAIL-mediated cell death receptor pathways. Moreover, combination treatment with CAP and AuQDs suppressed the motility and sphere-formation of brain cancer cells, which are recognized indicators of cancer aggressiveness. Taken together, the application of AuQDs can improve the efficiency of CAP against brain cancer cells, posing an excellent opportunity for advancing the treatment of aggressive glioblastomas.

## 1. Introduction

Cancer has become a leading global threat, and its burden is set only to increase in the coming years owing to population growth and lifestyle trends. With an estimated 18.1 million cases and 9.6 million deaths in 2018, cancer has been an increasingly pressing health and economic issue. Confronting cancer has gained the utmost attention in the field of biomedicine. Throughout the years, growing evidence clearly indicates that anticancer drugs can induce apoptosis via their cytotoxic effects. Gold quantum dots (AuQDs) are zero-dimension gold-based nanomaterials with tiny particle sizes of 2 to 10 nm that exhibit intriguing optical, electrical, and chemical properties due to their quantum confinement effect. Owing to their advantages, AuQDs have been utilized for biomedical applications, including cancer therapy. In our earlier studies, we observed that AuQDs suppress cancer invasiveness and stemness, thus enhancing the anti-tumorigenic effect of drugs [1]. Nevertheless, in general, the usage of AuQDs for cancer treatment remains challenging owing to the limited uptake of nanoparticles through the cell membrane. To tackle this problem, combinations of nanoparticles with cold plasma have recently emerged as a new potential therapy method [2,3,4,5], especially gold-based nanomaterials due to their unique properties [6,7,8,9,10,11]. For instance, He et al. reported that non-thermal plasma can temporarily increase cell membrane permeability to enhance endocytosis to uptake the gold nanoparticles, thus producing synergistic cytotoxicity to the target cancer cells [12]. The cold plasma-assisted nanoparticle-enhanced uptake can be due to enhanced endocytosis and trafficking to the lysosomal compartment as well as temporarily increased membrane permeability (pore formation or leaky membrane or passive diffusion) due to plasma treatment [1,4,12]. Our previous study also showcased a novel strategy of using cold plasma and PEG-coated gold nanoparticles to inhibit the PI3K/AKT signaling pathway, thus preventing the epithelial–mesenchymal transition and development of tumor [13]. In this study, to further extend this research direction, we presented a new combination treatment with AuQDs and plasma against glioblastoma cells.

The mechanisms by which various nanoparticles exert these cytotoxic effects are not fully understood. Apoptotic cell death classically involves two distinct pathways: the death receptor-mediated extrinsic mechanism and the Bcl-2-regulated intrinsic pathway [14,15,16] Members of the death receptor family include Fas, tumor necrosis factor (TNF)-related apoptosis-inducing ligand (TRAIL) death receptors 4 and 5 (DR4 and DR5), and tumor necrosis factor receptor 1 (TNFR1) [17,18]. Furthermore, the effect of cell death receptors on apoptotic signaling has not been widely reported by several anticancer treatments, and it is largely unknown whether AuQDs can induce death receptor signaling pathways in a combined treatment with plasma, which contributes to decrease the malignant progression of aggressive brain cancer phenotypes.

In this study, we aim to investigate the role of AuQDs and cold plasma against brain cancer cells, as well as their basic mechanism. We attempt to establish a novel cancer treatment method through plasma-assisted enhanced delivery of AuQDs. The non-thermal soft jet plasma device of the Plasma Bioscience Research Center was prepared and characterized. The soft jet plasma source was used in combination with quantum dots for assisted delivery, and its effect on the growth and invasiveness of malignant glioblastoma cells was evaluated. These investigations offer an exciting new therapeutic strategy for the treatment of resistant cancers.

## 2. Results and Discussion

### 2.1. Cold Atmospheric Plasma (CAP) Soft Jet Device and AuQD Characterization

**Figure 1:**
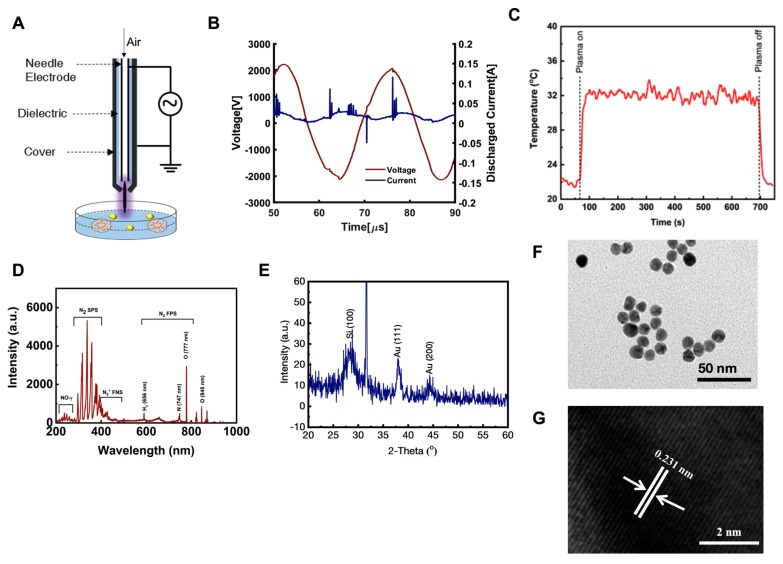
Cold atmospheric soft jet plasma (CAP) and gold quantum dots (AuQDs) characterization. (**A**) Schematic, (**B,C**) current-voltage and temperature profile, and (**D**) optical emission spectroscopy (OES) spectrum of the soft plasma jet. (**E**) X-ray diffraction pattern (XRD) of prepared AuQDs, deposited on silicon wafer (Si 100), (**F**) low-resolution transmission electron microscopy (TEM) analysis of GODs (~5 nm size), and (**G**) high-resolution (HR-TEM) images of AuQDs, which depict the difference between two lattice fringes and crystalline character of prepared products, which are ~0.231 nm.

Figure 1A shows the schematic of the soft plasma source, which consisted of a needle-type powered electrode inside a cylindrical glass tube protected by a 3D-printed plastic cover. Natural air was used as a feeder gas at a flow rate of l.0  lpm. The discharge duty ratio was set at ca. 11% (on time = 10 ms, off time = 82 ms). The current-voltage profile of the plasma on time is provided in Figure 1B. Plasma was generated at a high frequency of 42 kHz with a maximum voltage of 2.2 kV and a maximum discharge current of 100 mA. The temperature of the plasma plume was measured by a Luxtron m600 fluoroptic thermometer to be about 32 °C. (Figure 1C). The optical emission spectrum of the µ-DBD plasma source was recorded by an HR4000CG-UV-NIR (Ocean Optics, Dunedin, FL, USA). Figure 1D shows the optical emission spectroscopy spectrum of the soft plasma jet. We observed the emission of various reactive oxygen species (RNS) and reactive nitrogen species (ROS) as a result of using air as the feeding gas. We detected the emission of the N_2_ first positive system (N_2_ FPS) at 296.88, 316.71, 337.83, 358.63, 376.97, 381.21, 392.21, 392.49, and 400.82 nm; N_2_ s positive system (N_2_ SPS) at 428.04 and 500.77 nm; N_2_^+^ first negative system (N_2_^+^ FNS) at 590.02 747.93, 821.65, and 869.1 nm; and NO-γ band in the range of 200 to 250 nm. Atomic oxygen emission lines were detected at 777 and 845 nm. In addition, there was a weak emission of Hα at 656 nm.

The particle size, phase, and crystallinity of the prepared colloidal solution was examined through the X-ray diffraction pattern (XRD) pattern and the results are presented in Figure 1E. XRD pattern was evaluated by drop-casting the sample on a silicon wafer, followed by drying with gentle heat and fixation in a sample holder. In the obtained spectrum, we identified three different peaks: two peaks at 38.17 and 44.45, which were related to the AuQDs, and one peak at 31.7, which indicated the used silicon wafer/substrate. The denoted peaks resembled and matched the available Joint Committee on Powder Diffraction Standards card No. 04-0784 with face-centered cubic geometry. The broad peak width signified that the size of the prepared particles was very small. The estimated particle diameter of AuQDs was analyzed using the well-known Scherrer equation, as described previously [19]. The structural detail was investigated via HR–TEM and selected area electron diffraction pattern. As described in Section 4, TEM images were captured through deposition of colloidal gold solution on carbon-coated copper grids, and the images are shown in Figure 1F. The images showed that very small particles were sprinkled on the surface. Once their morphology was studied in detail, the average size of an individual particle was determined to be 4 to 5 nm (Figure 1F). The obtained image also revealed that each particle appeared to be spherical in shape, without forming aggregates with other structures. Crystallinity was also confirmed with HR–TEM, and a representative image is presented in Figure 1G. The distance between two fringes were approximately 0.231 nm, as estimated by HR–TEM, and this was equal to the FCC structures of gold particles [20].

### 2.2. AuQDs and CAP Diminish Cancer Cell Viability through Long-Term Inhibition of Cell Proliferation

**Figure 2:**
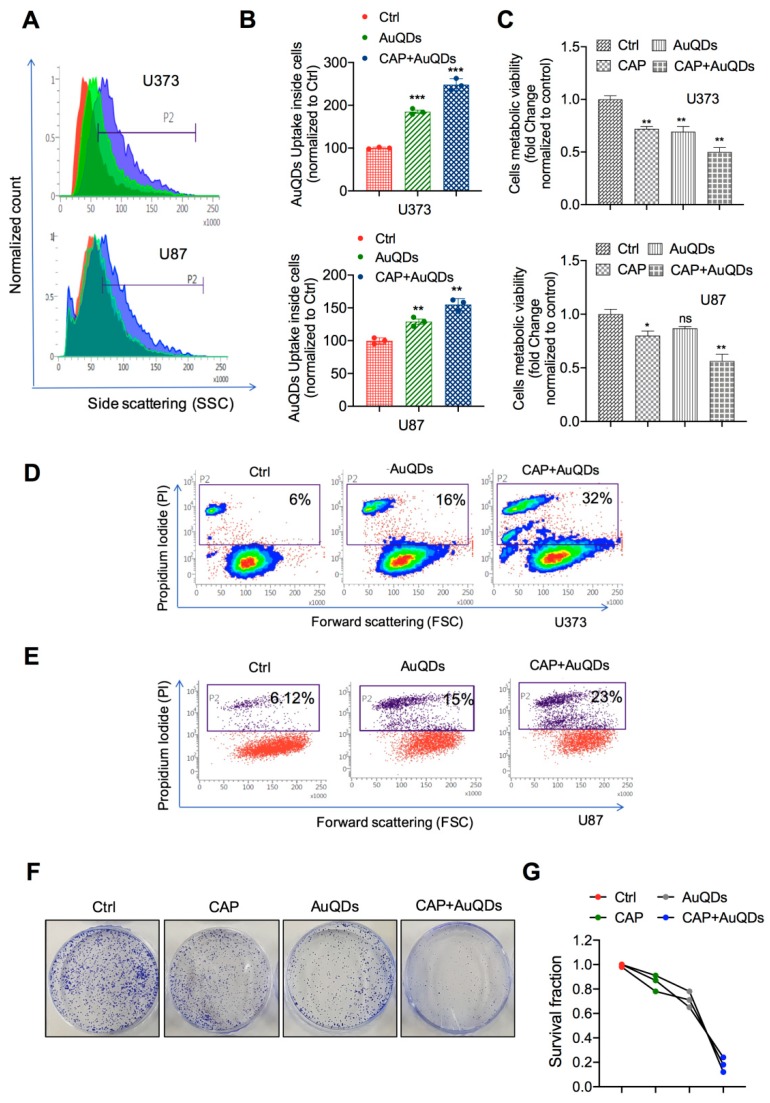
CAP and AuQDs co-treatment decreases proliferation of glioblastoma cancer cells. (**A**) AuQDs uptake analysis was performed in U373 and U87 brain cancer cells upon indication panels (AuQDs (25 nM) and CAP (200 s) treatment and measured by flow cytometry side scattering intensity (SSC-A) histograms after 24 h incubation time. (**B**) Quantification of FACS analysis are shown in graph. (**C**) Alamar blue viability test was done in U373 and U87 cells after AuQDs (25 nM) and CAP (200 s) treatment at 48 h. (**D,E**) Cell death analysis detected by propidium iodide (PI) was performed in untreated and AuQDs and CAP co-treated U373 and U87 cells, respectively. (**F,G**) Clonogenic formation assay in U373 brain cancer cells after only AuQDs (25 nM) or CAP (200 s) treatment and combination treatment. * *p* < 0.05, ** *p* < 0.001, and *** *p* < 0.0001; determined by two-tailed Student’s *t*-test (95% confidence interval).

To enhance the survival of patients with glioblastoma, alternative treatments are widely studied, including antibody–drug conjugate-based and nanomaterial-based therapies [21,22]. In our previous studies, we have used CAP for the treatment of various cancer cells, including brain cancer cells [23,24]. In particular, we have recently reported that low doses of plasma in the presence of PEG-coated non-thermal plasma inhibited the progression of solid cancer cells [13]. On the basis of these studies, we examined the effect of the AuQDs we synthesized in a combination with plasma treatment. We have already reported the specificity of AuQDs in glioblastoma cells [1]; therefore, we further investigated whether AuQDs improve the efficiency of plasma treatment against glioblastoma cell progression. To this end, we tested the effect of AuQDs combined with soft jet plasma. Prior to cellular phenomenon analysis, we examined the cellular uptake of AuQDs by brain cancer cells. We observed a great increase in the cellular uptake of AuQDs by U373 and U87 cells; however, this effect was more prominent in the AuQD and plasma-treated groups than in the groups treated with AuQDs alone (Figure 2A,B). This finding indicated that this increase was caused by the plasma, which generated short-long lived species in the nanoparticles. Next, we investigated whether AuQDs alone or a combination of AuQDs with plasma exert cytotoxicity in U373 and U87 brain cancer cells (Figure 2C). These cells were treated with AuQDs (25 nM) alone or in combination with plasma (25 nM AuQDs + 200 s plasma) and incubated for 48 h. Our data showed that cellular viability significantly decreased in the groups treated with the combination compared to that in the groups treated with AuQDs alone or in the untreated groups in both cells (Figure 2C). Notably, propidium iodide (PI) analysis showed cell death rate of approximately 25%–30% after treatment with AuQDs and plasma under similar dose conditions; however, cell death rate was only approximately 16% after treatment with AuQDs alone in U373 and U87 cells (Figure 2D,E). This result was supported by the colony formation assay results (Figure 2F,G). These findings showed that intracellular AuQDs play an important role in plasma sensitivity by regulating the cell proliferation, survival, and death of cancer cells.

### 2.3. AuQDs and CAP Co-Treatment Induced Reactive Oxygen/Nitrogen Species (RONS) Suppresses Cell Growth

**Figure 3:**
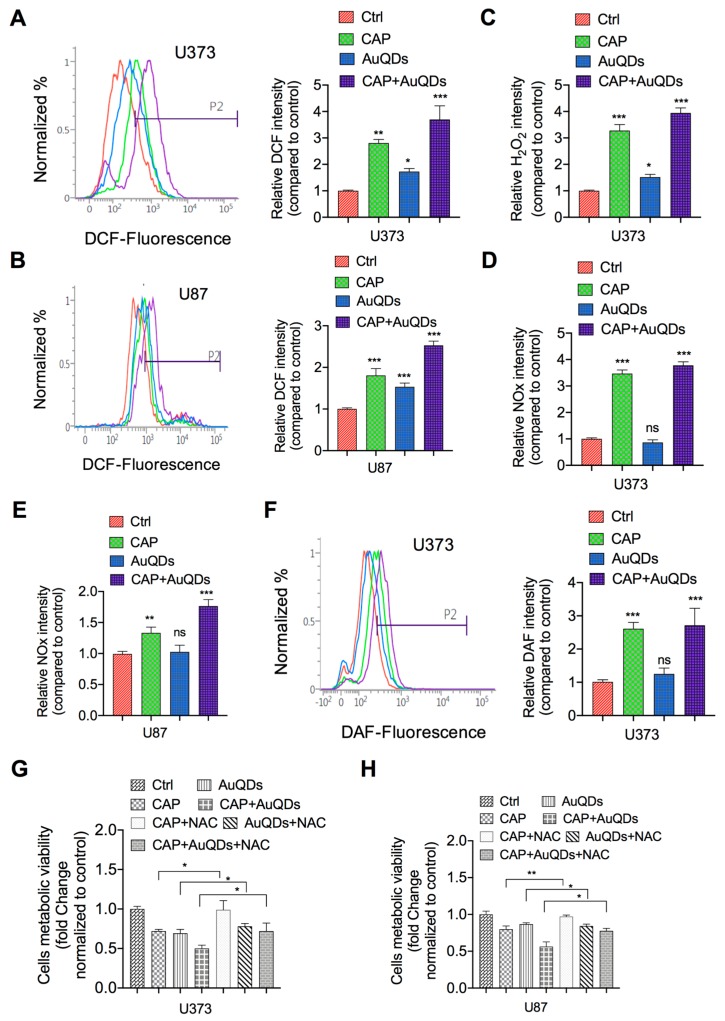
Analysis of reactive oxygen nitrogen species by CAP and AuQDs. (**A,B**) Intracellular ROS levels are detected by H2DCFDA (10 μM) fluorescent dye in CAP, AuQDs alone, or co-treated U373 and U87 brain cancer cells, respectively. Quantification of ROS are shown in graph. (**C**) Detection of H_2_O_2_ levels (cells including medium) in CAP, AuQDs alone, or co-treated U373 cells. (**D,E**) Measurement of nitrogen species (NOx) in CAP, AuQDs alone, or co-treated U373 and U87 cells, respectively, including medium by assay kit. (**F**) Detection of intracellular nitric oxide by DAF-DA (10 μM) fluorescent dye in similar treated groups using U373 cells (left panel). Quantification are shown in graph (right panel). (**G,H**) Analysis of cell viability of U373 and U87 cells, respectively, in presence or absence of N-acetyl cysteine (NAC 4 mM) among all groups as mentioned in indicated panels. All these RONS tests were performed at 12 h after indicated treatments; however, viability was tested after 48 h. NAC was pretreated at 6 hr before treatments. Treatment doses were similar under all the experiments. * *p* < 0.05, ** *p* < 0.001, and *** *p* < 0.0001; determined by two-tailed Student’s *t*-test (95% confidence interval).

The above results indicate the potential effect of co-treatment of AuQDs and plasma on suppressing cellular growth and proliferation in brain cancer cells. One of the main mechanisms involved in CAP on anti-cancer activity is reactive species [13,23]. We questioned whether any RONS factors are involved in this growth suppression. To this end, we studied the role of reactive species in the anti-glioblastoma effect by combination treatment of CAP and AuQDs. We checked relative levels of intracellular reactive oxygen species (ROS), reactive nitrogen species (RNS), H_2_O_2_ and NOx after treatment. Data show that the ROS level significantly increased after treatment by AuQDs, plasma, and combination treatment in U373 and U87 cells (Figure 3A,B). H_2_O_2_ levels also showed higher increase in combination treatment in U373 cells, as shown in Figure 3C. The enhanced reactive species levels are majorly synergistic in case of intracellular ROS and H_2_O_2_ levels by combination treatment. Moreover, the high ROS and H_2_O_2_ level in the co-treated U373 cells indicated that AuQDs could sensitize the cellular cytotoxicity of CAP. In addition, there is no synergistic effect observed in the case of intracellular RNS and NOx levels by combination treatment (Figure 3D–F). Our data show that AuQDs alone failed to induce the generation of RNS and NOx significantly in glioblastoma cells. According to the present findings, we can confirm that only plasma is the source of RNS for an anti-glioblastoma effect. To verify further, we also used the intracellular ROS scavenger N-aceyl cysteine (NAC) to check the effect of reactive species on viability after AuQDs and plasma treatment. The intracellular ROS scavenger NAC significantly counteracts anti-glioblastoma effect in AuQDs, CAP, and combination-treated U373 and U87 cells (Figure 3G,H). These data conclude that combination treatment of Au-QDs and plasma synergistically enhanced ROS and H_2_O_2_ for an anti-glioblastoma effect.

### 2.4. AuQDs and CAP Affect Cancer Cell Motility and Self-Renewal after Loss of Cell-to-Cell Contact Adhesion

**Figure 4:**
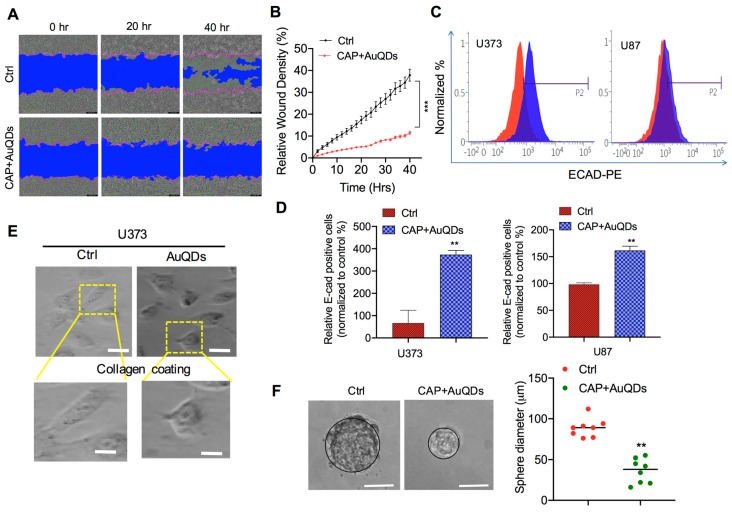
Dual treatment of CAP and AuQDs inhibits malignant ability of glioblastoma cancer cells. (**A**) Wound healing assay in U373 brain cancer cells upon indication panels (AuQDs (25 nM) and CAP (200 s) co-treatment after 20 h and 40 h measured by Sartorius Incucyte. (**B**) Wound density is shown in representative graph. (**C**) Measurement of E-cadherin positive cells in untreated and AUQDs-CAP co-treated U373 and U87 cells by flow cytometry. (**D**) Quantification of E-casdherin positive cells anlyzed by flow cytometry. (**E**) Morphological analysis of untreated and AuQDs–CAP co-treated U373 cells observed on collagen-coated surface. (**F**) Self-clonal formation assay in untreated and AuQDs–CAP co-treated U373 cells detected after 11 days in 96-well plate (left panel). Average sphere size (diameter) was calculated and represented as graph (right panel). Treatment doses were similar under all the experiments. Scale bar = 10 µm. * *p* < 0.05, ** *p* < 0.001, and *** *p* < 0.0001; determined by two-tailed Student’s *t*-test (95% confidence interval).

To further assess the cytocompatibility of AuQDs and plasma in human cancer cells, the wound-scratch assay was performed to examine the migration of cancer cells. Previous research reported that malignant cancer cells can facilitate tumor migration. Cell migration is itself is a highly dynamic process that includes attachment loss and changes in cell cytoskeleton. A wound healing assay showed a remarkable difference between brain cancer cells treated with AuQDs and plasma and the untreated control (Figure 4A,B). Cell-to-cell contact was enhanced, as indicated by E-cadherin expression in U373 and U87 brain cancer cells (Figure 4C,D). In addition, elongated U373 cells regained their original phenotype after co-treatment with AuQDs and plasma (Figure 4E). The decrease in cell migration could be due to compact cell-to-cell adhesion induced by treatment with plasma and AuQDs, which blocked cancer cell movement. It has been claimed that the development of resistant cancer stem cells is associated with cell migration and invasion, which is usually responsible for tumor relapse [25,26]. In this study, U373 brain cancer cells cultured in serum-free sphere culture media lost their ability for self-renewal after treatment with AuQDs and plasma, as suggested by the clonal formation (Figure 4F). These results indicated that treatment with AuQDs and plasma possibly impaired the malignancy of U373 cells.

### 2.5. Increased Fas Expression Induces Casp8 Accumulation by AuQDs and CAP Treatment

**Figure 5:**
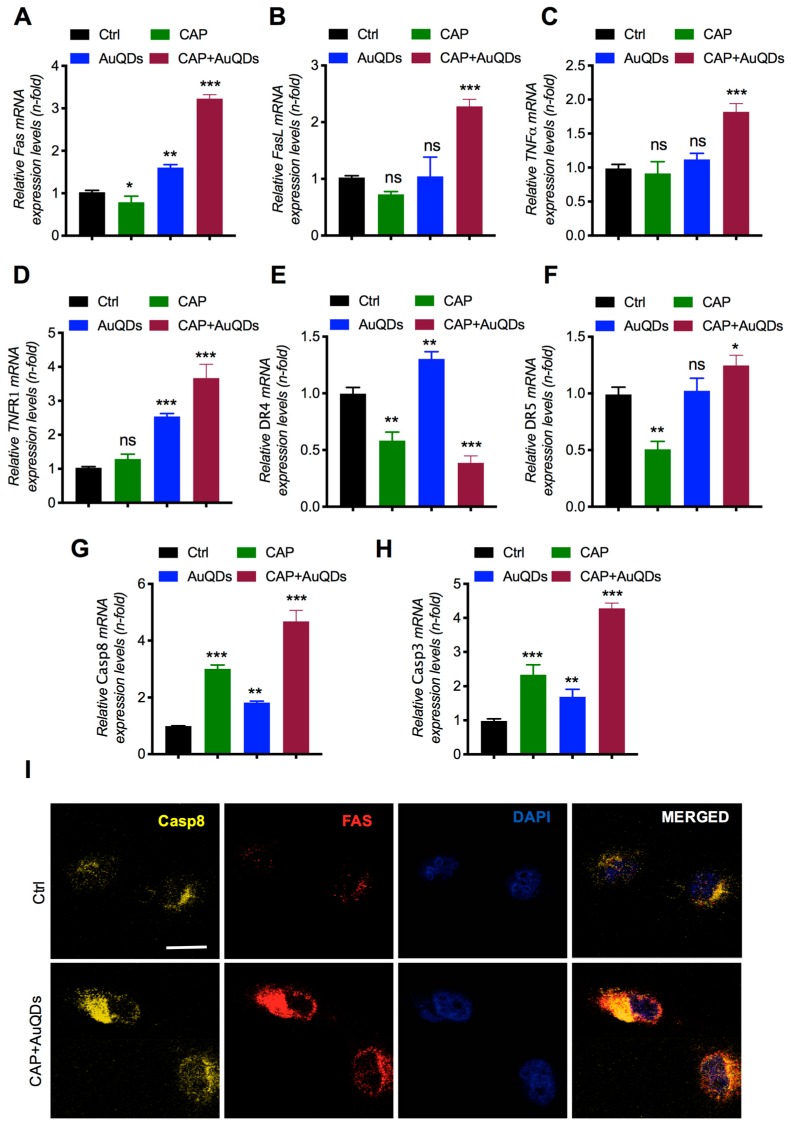
Effect of CAP and AuQDs on cellular growth was mediated by death receptor pathways in glioblastoma cancer cells. (**A–H**) q-RT PCR analysis of cell death receptor gene expression such as Fas, FasL, TNF-a, TNFR1, DR4, DR5, Casp8, and Casp3 in U373 brain cancer cells upon AuQDs (25 nM) and CAP (200 s) co-treatment after 48 h. (**I**) Immunofluorescence analysis of Casp8 and Fas in U373 cells upon AuQDs (25 nM) and CAP (200 s) co-treatment after 48 h. β-actin was used as a normalized control. Scale bar = 100 µm. * *p* < 0.05, ** *p* < 0.001, and *** *p* < 0.0001; determined by two-tailed Student’s *t*-test (95% confidence interval).

Our results so far showed that combination treatment with AuQDs and plasma induced death in brain cancer cells. We next sought to determine the signaling mechanism of AuQDs and plasma-induced cytotoxicity in brain cancer cells. To this end, we examined whether the AuQDs and plasma dual treatment alter the levels of proteins involved in apoptosis in brain cancer cells. Apoptosis is induced by the death ligand TNF, Fas ligand (FasL), or TRAIL. It has been suggested that apoptosis is initiated by the binding of FasL to the Fas receptor or of TRAIL to either DR5 or DR4 receptors [27]. This leads to the direct enrollment of FADD, allowing the binding of procaspase-8 to the intracellular death-inducing signaling complex. Considering all these findings, we examined the expression of Fas, FasL, TNFR1, TNFa, DR4, and DR5, as well as Casp3 and Casp8. Our data showed that the expression of these markers was noticeably enhanced in the groups treated with AuQDs and plasma compared to the groups treated with AuQDs alone or in the untreated groups (Figure 5A–H). Immunofluorescence analysis also confirmed that increased Fas expression also enhanced Casp8 expression in U373 cells treated with AuQDs and plasma compared to the untreated groups (Figure 5I). These results revealed the AuQDs and plasma co-treatment exerted a cytotoxic effect on brain cancer cells through death receptor-mediated pathways.

## 3. Materials and Methods

### 3.1. Synthesis of Gold Quantum Dots (AuQDs)

Fold quantum dots were generated using chloroauric acid trihydrate (HAuCl_4_•3H_2_O) and the reducing agent trisodium citrate dihydrate (N_3_C_6_H_5_O_7_, 1%), which were acquired from Aldrich Chemical Co., Ltd. and used without any further purification. For this experiment, a very small amount of ~1 mM HAuCl_4_•3H_2_O was dissolved in 100 mL of deionized water; the pH of the solution reached 2.81. To this gold chloride solution, 1% trisodium citrate dihydrate (N_3_C_6_H_5_O_7_, 3 mM) was added. The pH of the solution was confirmed to increase to 7.75. The pinkish-colored colloidal solution was stirred continuously for 10 to 15 min. Once the stirring was completed, the solution was transferred to a refluxing pot and refluxed to their boiling temperature for 15 min. When the color of the solution went dark and deep red, the refluxing was stopped and the solution was cooled at room temperature. The obtained colloidal solution was subjected to structural and chemical analyses [20].

### 3.2. Characterization of Colloidal Solution of Gold

Structural/morphological analysis was conducted by high-resolution transmission electron microscopy (HR–TEM) (200 kV, Jeol JSM 2010; Hitachi, Tokyo, Japan). The pinkish-colored solution was initially sonicated for approximately ~10 to 15 min in a specialized bath sonicator (40 kHz; Cole Parmer, Vernon Hills, IL, USA). Once the sonication was completed, the carbon-coated copper grid was dipped to this solution for 2–3 min. Next, the copper grid was heated on a hot plate with gentle heating and then fixed in a sample holder for morphological analysis [20]. The crystallinity of the prepared gold colloid was checked via XRD analysis (Rigaku, Tokyo, Japan) with CuKα radiation (λ = 1.54178Å) in the range of 20–80º with 6º/min scanning speed. The sample for XRD analysis was prepared using a clean silicon substrate. The prepared gold solution was drop-casted on to the silicon substrate and then dried at room temperature. The dried silicon subtracted with sample was then fixed in a sample holder of the XRD instrument and analyzed.

### 3.3. Cell Culture, Antibodies, and Reagents

U373MG and U87 cells (grade III, glioblastoma multiforme) were purchased from Korean Cell Line Bank (Seoul, Korea) and then cultured and stored according to Korean Cell Line Bank standards. Briefly, U373MG cells were cultured in DMEM (cat# LM001-05; Welgene, Gyeongsangbuk-do, Korea) supplemented with 10% fetal bovine serum, 100 U/mL of penicillin, and 100 μg/mL of streptomycin and maintained in a humidified incubator at 37 °C with 5% CO_2_. The cells were sub-cultured every two to three days. For glioblastoma sphere cultures, media were prepared using EGF, FGF, and B27 (1X) in serum-free DMEM, as described previously [13]. Antibodies specific to Fas (sc-8009) and Casp8 (sc-81656) were purchased from Santa Cruz Biotechnology, Inc. All PCR primers were designed and purchased from DNA Macrogen, Seoul, Korea. The primer sequences used are mentioned in Table S1.

### 3.4. Cell Viability Assay

Alamar blue dye (DAL1025; Thermo Fisher Scientific, Waltham, MA, USA) was used to assess the viability of U373 cells after treatment with AuQDs and plasma. To this end, U373 cells were seeded at a density of 5 × 10^4^/mL cells per well in 24-well cell culture plates. Briefly, each set contained a control (untreated) and treatment groups (AuQDs alone and/or in combination with air soft jet plasma at 200 s). For the combination treatment, cells were exposed for 200 s with air soft jet plasma after 5 h of treatment with 25 nM AuQDs. In a different experiment, cellular viability was assessed for U87 and U373 cells post-treatment with AuQDS alone, plasma alone, or in combination (AuQDs-plasma) in presence/absence of N-acetylcysteine (4 mM, Sigma-Aldrich, Seoul, Korea), a ROS scavenger. Alamar blue conversion was measured by monitoring the fluorescence, as described in our previous report [28].

### 3.5. Cell Death Assay

Death of U373 cells after treatment with AuQDs alone and in combination with plasma exposure was determined by evaluating the PI uptake of the cells. PI (Sigma Aldrich, Seoul, Korea) was prepared in PBS (Gibco, Langley, OK, USA) at a concentration of 50 ng/mL as a working stock solution. For PI analysis, 2 × 10^5^ cells/well were seeded in triplicates in 30-mm dishes. After 24 h of treatment as described above, the cells were washed with PBS and harvested using 0.25% trypsin–EDTA (Cat #SH30042.01; HyClone, Logan, UT, USA), followed by the addition of medium supplemented with 10% fetal bovine serum to neutralize the effects of trypsinization. The cells were subsequently centrifuged to obtain a pellet. The pellet was resuspended in PBS containing PI and subjected to flow cytometry analysis using FACSVerse (BD Biosciences, San Jose, CA, USA).

### 3.6. Cellular Uptake Analysis

For measurement of cellular uptake, the cells were exposed to AuQDs, washed twice with PBS, trypsinized, centrifuged at 1000 rpm for 3 min, and further resuspended in PBS. The side scattering parameter was used to measure the intracellular NP uptake of the cells, as described previously [1].

### 3.7. Wound Healing Assay

To analyze cell migration, the wound healing scratch assay was performed on untreated control cells and cells treated with AuQDs and soft jet plasma. The cells were cultured to 100% confluence in 96-well plates and serum-starved after 8 h of seeding until the end of the experiment. At 95% confluence, a scratch wound was made on the cell culture using a Sartorius wound maker, followed by treatment. Air soft jet plasma (200 s)-treated medium was mixed with 25 mM AuQDs and used for treatment. After the treatment, the plates were incubated in a Sartorius Incucyte Korea, and cellular migration was scanned and captured every 2 h using Sartorius Incucyte software (https://www.essenbioscience.com/en/products/software/incucyte-base-software/) After 2 days, image scanning was stopped for data analysis using Incucyte software.

### 3.8. RNA Extraction and Real-Time PCR

Briefly, RNA from untreated cells and cells treated with AuQDs and jet plasma was manually extracted using Trizol reagent (Invitrogen, Seoul, Korea). All reactions were performed using a KAPA SYBR FAST qRT-PCR kit (KAPA Biosystems, Wilmington, MA, USA) in a Rotor Gene Q thermocycler (Qiagen, Seoul, Korea), and the results were expressed as the fold change.

### 3.9. Clonogenic, Collagen Coating, and Self-Renewal Assay

U373 cells were harvested after the treatments, incubated for 48 h, and replated (500 cells/well) in a six-well plate. These sample plates were then further incubated for an additional 2–3 weeks at 37 °C for growth analysis. Afterwards, the cells were fixed using 70% EtOH and stained with 0.5% crystal violet. Colonies were counted using a standard colony counter. Plating efficiency (PE) and surviving fractions were calculated according to a previously described protocol [29]. For collagen coating analysis, collagen-coated plates (Corning^®^; BioCoat™, Corning, NY, USA) were purchased and used for seeding the cells after treatments. Morphological analysis was performed after 48 h of incubation with the treatments. For self-renewal analysis, glioblastoma sphere cells were seeded into 96-well plates at a density of 1 cell per well. At the next day, each well was visually checked to detect the presence of a single cell. The single-cell clones were grown, and clone formation was monitored on days 1 and 15. Clone size was analyzed using a bright-field phase contrast microscope with Motic Images Plus 2.0, Hong Kong [30].

### 3.10. Reactive Oxygen Nitrogen Species (RONS) Detection

Briefly, fluorescent dyes, H2DCFDA and DAF-FM-diacetate (Molecular probes, Invitrogen, Waltham, MA, USA), were used to detect free radicals of ROS and RNS, respectively. To this end, U373 and U87 cells cultured in 30-mm dish were given treatments for AuQDs and plasma separately or in combination, followed by incubation for 24 h. Cells were then trypsinized, washed with PBS and incubated with 10 μM of H2DCFDA and DAF-FM dyes for 40 min at 37 °C in dark. RONS generation was assessed and analyzed using FACS Suite software (BD FACSverse). In a different experiment setup, the concentrations of H_2_O_2_, and NOx (NO^-2^ and NO^-3^) were measured. For this, cells were seeded at a density of 5 × 10^4^/mL cells per well in 24-well cell culture plates and followed with the treatment as mentioned above. For H_2_O2 and NOx measurement, a quantichrome assay kit (DIOX, Biassay System, Highland, UT, USA) and a nitric oxide colorimetric assay kit (K262–200, BioVision, USA) were used in accordance to the manufacturers’ protocols, respectively. Fluorescence and absorbance were measured using the plate reader (SynergyTM HT, BioTek Instruments, Inc., city, Winooski, VT, USA).

### 3.11. Immunofluorescence

To visualize the expression levels of Casp8 and Fas, treated cells were fixed with 4% paraformaldehyde and permeabilized with 0.1% Triton X-100 in PBS. Subsequently, the cells were incubated with unconjugated Fas antibody (1:200) or casp8 antibody (1:200) in a blocking buffer (PBS with 1% BSA and 0.1% Triton X-100) at 4 °C overnight. Stained cells were visualized using Alexa Fluor 488 or PE (Invitrogen). Cell nuclei were stained with 4,6-diamidino-2-phenylindole (Sigma Aldrich) and visualized using a fluorescence microscope (Olympus IX71, Tokyo, Japan).

### 3.12. Statistical Analysis

Experimental data are expressed as the mean ± SD of triplicates. Student’s *t*-tests were performed and statistical differences between groups were analyzed. The differences were considered statistically significant when the *p*-value was lower than 0.05 (* *p* < 0.05, ** *p* < 0.01, *** *p* < 0.001).

## 4. Conclusions

In conclusion, these results determine in vitro cytotoxicity of AuQDs and plasma co-treatment on brain cancer cells based on decreased cell growth and induced apoptotic cell death. These treatments also induced morphological changes associated with malignant type. AuQDs and plasma induced cell apoptosis through death receptor pathways such as Fas, TNFR1, and the DR5 and DR4 receptor-mediated extrinsic pathway in brain cancer cells. Taken together, our results provide evidence that AuQDs can enhance the efficacy of plasma through activation of caspases with low concentration dose. Also, this dual approach can reduce malignancy, as observed by improvement of cell–cell contact adhesion and this represents a rationale for the use of AuQDs as an anticancer agent for improving target drug delivery systems using plasma. Previous in vivo studies have suggested that the administration of plasma treated medium supplemented with AuQDs could invoke dual cytotoxicity in mice. Nevertheless, further studies using clinically relevant animal models and human efficacy and safety studies are required to explore the therapeutic potential of plasma-assisted AuQD delivery against cancer.

## Supplementary Materials

The following are available online at https://www.mdpi.com/2072-6694/12/2/457/s1, Table S1: List of primer sequences used in the study.
