# Supplementary material for: Cold Atmospheric Plasma and Gold Quantum Dots Exert Dual Cytotoxicity Mediated by the Cell Receptor-Activated Apoptotic Pathway in Glioblastoma Cells
Source: Cancers (Basel). 2020 Feb 16;12(2):457. doi: 10.3390/cancers12020457 (PMC7072464; doi:10.3390/cancers12020457)
Supplement: Supplementary file 1 [file cancers-12-00457-s001.pdf]

Cold atmospheric plasma and gold quantum dots exert dual cytotoxicity mediated by the cell receptor-activated apoptotic pathway in glioma cells

Nagendra Kumar Kaushik, Neha Kaushik, Rizwan Wahab, Pradeep Bhartiya, Nguyen Nhat Linh, Farheen Khan, Abdulaziz A. Al-Khedhairy and Eun Ha Choi

Supplementary Information

Table S1. List of primer sequences used in the study.

Gene Name	Primer Sequence (5′-3′)	Product size (bp)
**Fas**	TGCCCAAGTGACTGACATCA (left)<br>CATCCCCATTGACTGTGCAG (right)	164
**TNFa**	ACCTCCTCTCTGCCATCAAG (left)<br>ATCCCAAAGTAGACCTGCCC (right)	187
**FasL**	GTCCAACTCAAGGTCCATGC (left)<br>TTGTTGCAAGATTGACCCCG (right)	156
**DR5 (TNFRSF10B)**	CAGACTTGGTGCCCTTTGAC (left)<br>TTGGCAAGTCTCTCTCCCAG (right)	208
**TNFR1**	CGGTGACTGTCCCAACTTTG (left)<br>AAACGCACTGTCTAGGCTCT (right)	163
**DR4 (TNFRSF10A)**	CACTTTCGTCTCTGAGCAGC (left)<br>CAGAGTCTCAGTGGGGTCAG (right)	172
**Casp3**	TGAGCCATGGTGAAGAAGGA (left)<br>GCACAAAGCGACTGGATGAA (right)	186
**Casp8**	AGGAGCTGCTCTTCCGAATT (left)<br>TGTCCATTCAACCCACACCT (right)	168
